# Halal Slaughter of Livestock: Animal Welfare Science, History and Politics of Religious Slaughter

**DOI:** 10.1017/awf.2024.18

**Published:** 2024-04-11

**Authors:** Claire White

**Affiliations:** National Farmers Union, Kenilworth

As the second published title for the author in an increasingly diverse repertoire, this book should be regarded as a formative exploration into the technical aspects of all slaughter practices, and as a contemporary discussion of religious slaughter in modern food systems. Both topics are individually complex yet clearly described in the book, and their fundamental interdependency explored through the examination of halal slaughter practices from across the globe. Approached as a holistic guide, capturing both the historic and contemporary position, it is wholly relevant to readers from all backgrounds – of any faith or none. This meets the author’s stated aim of informing all stakeholders, including consumers, veterinarians, policy-makers, religious authorities and industry colleagues.

As a concise work, covering an especially complex area of animal welfare and ethics, the book is sensibly divided into four chapters which independently address the fundamental aspects of halal slaughter. These are 1) the science of conscious perception and death, 2) impact of preslaughter handling on welfare, 3) religious slaughter, and 4) sociopolitical aspects of religious slaughter. Each chapter is written in the style of a literature review, beginning with an abstract of key points and concluding with a comprehensive reference list to support the concepts discussed. Readers unfamiliar with scientific writing should not be deterred however, since the author is extremely comfortable in employing non-technical language wherever possible and using explanatory figures to illustrate more complex points outside the main text. Consequently, the book can be readily used as a reference text for technical and theological enquiries by readers of any level, as well as a narrative account of the evolution of contemporary halal slaughter practices, and their wider reception as a prominent aspect of the Islamic religion. The final chapter succeeds in delivering a conclusion to this narrative, as well as considering the relevance of non-faith food concerns – including veganism, and climate crisis – to halal principles, reflecting current secular and societal interests.

Of note, the exploration of conscious perception and death is an essential foundation and of particular relevance to the later discussions, regarding slaughter practices. These discussions require an understanding of how beliefs surrounding the states can influence slaughter practices and their acceptance by faith and non-faith stakeholders. The subject is complex, but the author explains concepts simply and with the aid of explanatory figures which can be readily referred to when revisited in later chapters. Some readers more familiar with the subject matter may have hoped for more context to be provided as to how practices vary across the globe, since these are influenced by domestic legislation, societal expectations, cultural beliefs, and market requirements. Equally, those seeking to understand the practical challenges or potential solutions to innovation and advancements in contemporary halal slaughter may need to pursue further dialogue with the author. The author rightfully recognises the contributions of welfare charities such as The Humane Slaughter Association and Eyes on Animals to such advancements and a wider understanding of animal welfare by Muslims, as well as the nascent role of farm assurance in halal production systems.

The chapters are generally well balanced in their approach to the subject in question, and sufficiently concise for them to be read in one sitting. However, some may find chapter four, which covers religious slaughter, a more lengthy and challenging read, since it covers both the historical relevance of stunning equipment development and the origins of halal slaughter. The rationale behind incorporating both these subjects into one larger chapter is understandable, since it reminds the reader that there are many parallels between the evolution of both faith and non-faith slaughter practices. A consonant history and future is one of the key messages which the author is seeking to convey through the overarching narrative and should be recognised by readers as the foundation of a unified approach to the advancement of welfare at slaughter. This objective is echoed in the foreword, written by Rizvan Khalid, a highly regarded Muslim meat industry expert, and the Animal Welfare series preface, written by series editors Moira Harris and Clive Phillips. The contributors all recognise that a unified industry, where animal welfare is at the forefront of all advancements, and where market requirements or preferences are recognised and respected, will not only help the agri-food sector maximise the opportunities, but ultimately bring about greater understanding and consensus.

